# The ubiquitin ligase TRIM32 promotes the autophagic response to Mycobacterium tuberculosis infection in macrophages

**DOI:** 10.1038/s41419-023-06026-1

**Published:** 2023-08-05

**Authors:** Alessandra Romagnoli, Martina Di Rienzo, Elisa Petruccioli, Carmela Fusco, Ivana Palucci, Lucia Micale, Tommaso Mazza, Giovanni Delogu, Giuseppe Merla, Delia Goletti, Mauro Piacentini, Gian Maria Fimia

**Affiliations:** 1 Department of Epidemiology, Preclinical Research and Advanced Diagnostics, National Institute for Infectious Diseases IRCCS ‘L. Spallanzani’, Rome, Italy; 2 Division of Medical Genetics, Fondazione IRCCS Casa Sollievo della Sofferenza, 71013 San Giovanni Rotondo, Italy; 3 Dipartimento di Scienze Biotecnologiche di Base, Cliniche Intensivologiche e Perioperatorie-Sezione di Microbiologia, Università Cattolica del Sacro Cuore, 00168 Rome, Italy; 4 Dipartimento di Scienze di Laboratorio e Infettivologiche, Fondazione Policlinico Universitario “A. Gemelli”, IRCCS, 00168 Rome, Italy; 5 Bioinformatics laboratory, Fondazione IRCCS Casa Sollievo della Sofferenza, 71013 San Giovanni Rotondo, Italy; 6 Mater Olbia Hospital, 07026 Olbia, Italy; 7 Laboratory of Regulatory & Functional Genomics, Fondazione IRCCS Casa Sollievo della Sofferenza, San Giovanni Rotondo, Foggia, 71013 Italy; 8 Department of Molecular Medicine & Medical Biotechnology, University of Naples Federico II, Naples, 80131 Italy; 9 Department of Biology, University of Rome ‘Tor Vergata’, Rome, Italy; 10 Department of Molecular Medicine, University of Rome “La Sapienza”, Rome, Italy

**Keywords:** Macroautophagy, Innate immunity

## Abstract

*Mycobacterium tuberculosis* (Mtb) is known to evade host immune responses and persist in macrophages for long periods. A mechanism that the host uses to combat Mtb is xenophagy, a selective form of autophagy that targets intracellular pathogens for degradation. Ubiquitination of Mtb or Mtb-containing compartments is a key event to recruit the autophagy machinery and mediate the bacterial delivery to the lysosome. This event relies on the coordinated and complementary activity of different ubiquitin ligases, including PARKIN, SMURF1, and TRIM16. Because each of these factors is responsible for the ubiquitination of a subset of the Mtb population, it is likely that additional ubiquitin ligases are employed by macrophages to trigger a full xenophagic response during Mtb infection. In this study, we investigated the role TRIM proteins whose expression is modulated in response to Mtb or BCG infection of primary macrophages. These TRIMs were ectopically expressed in THP1 macrophage cell line to assess their impact on Mtb replication. This screening identified TRIM32 as a novel player involved in the intracellular response to Mtb infection, which promotes autophagy-mediated Mtb degradation. The role of TRIM32 in xenophagy was further confirmed by silencing TRIM32 expression in THP1 cells, which causes increased intracellular growth of Mtb associated to impaired Mtb ubiquitination, reduced recruitment of the autophagy proteins NDP52/CALCOCO2 and BECLIN 1/BECN1 to Mtb and autophagosome formation. Overall, these findings suggest that TRIM32 plays an important role in the host response to Mtb infection through the induction of autophagy, representing a promising target for host-directed tuberculosis therapies.

## Introduction

*Mycobacterium tuberculosis* (Mtb), the etiological agent of tuberculosis (TB), has evolved a wide variety of strategies to survive within host macrophages [1]. In most cases, the host immune system can contain Mtb infection in a non-sterilizing manner thanks to the contribution of the innate and adaptive immune responses, which results in an infection that can persist whole life time [2, 3]. TB disease occurs in 3–10% of patients, in which bacterial replication overcomes the immune defenses resulting in an increase in bacterial burden and tissue damage [4]. Recent evidence indicates that the spectrum from TB infection to TB disease is more complex, including a "continuum" of subclinical situations, which also contribute to Mtb transmission [5].

Molecular mechanisms that *Mtb* has developed to evade host innate immunity include cytosolic escape, restricted production of antimicrobial peptides, blockade of phagosome maturation and antigen presentation, inflammasome activation, and modulation of cell death and autophagy [6, 7]. Autophagy is a main catabolic process that guarantees cellular fitness by mediating turnover of intracellular components in both basal and stress conditions [8]. Materials to be degraded are sequestered in double-membrane vesicles called autophagosomes, which eventually fuse with lysosomes for degradation [8]. In addition to mediate self-digestion, autophagy contributes to immune defense during pathogen infection by delivering intracellular pathogens to the lysosomal compartment, a selective type of autophagy referred as xenophagy [9–12]. This event leads to both bacterial killing and the accessibility of pathogen components to innate and adaptive immune receptors [11]. In particular, an important role of autophagy in restricting Mtb replication in macrophages has been recently confirmed using both human and mouse models [13–15]. On the other hand, Mtb has evolved different strategies to modulate the autophagic activity through the ESX-1 secretion system and PE_PGRS and PE/PPE-containing proteins [7, 12, 16–18]. Interestingly, genetic variability of Mtb may account for a different ability to modulate the autophagy response in primary macrophages [19].

As for other types of selective autophagy, recognition of cargos in xenophagy is achieved through different types of autophagy adapters, which physically link cargos and autophagosomal proteins of the Atg8/LC3 family [20]. The best characterized type of autophagy adapters are those belonging to the sequestosome 1/p62-like receptors (SLR) family, which deliver ubiquitinated structures to autophagosomes, containing both ubiquitin-associated and LC3 interacting domains [21, 22]. In the case of Mtb infection, two ubiquitin ligases responsible for ubiquitination of Mtb-containing structures have been reported. The ubiquitin ligase Smurf1 transfers preferentially K48-linked Ub chains on Mtb, targeting it to the autophagosomes via the autophagy adapter NBR1 [23], while Parkin mediates K63-linked Ub chains, which are recognized by the p62/SQSTM1 and NDP52/ CALCOCO2 adapter proteins [24]. Intriguingly, host xenophagy is also triggered by ubiquitin recruited to Mtb in an ubiquitin ligase-independent manner by direct binding to the surface protein PE_PGRS29 [25].

Along with ubiquitin, autophagy adapters are recruited to Mtb by galectins, which bind to cytosolic glycan residues exposed by phagosomes damaged by the Mtb secretion systems, as shown for TAX1BP1 and Galectin-8 [26], and by ubiquilin-1, an ubiquitin-like, and ubiquitin-binding domain protein, which promotes ubiquitin, p62, and LC3 accumulation around Mtb in IFN-γ activated macrophages [27]. Interestingly, recent evidence showed that ubiquitination is also required for the membrane repair of vacuoles damaged by the *M. marinum*, a close relative of Mtb, to prevent its cytosolic escape through the recruitment of the ESCRT machinery and the nascent autophagosome vesicles [28].

How the autophagy core machinery responsible for autophagosome formation is activated and recruited to Mtb during infection remains less characterized. NEDD4, a HECT type of ubiquitin ligase, has been shown to increase BECLIN 1 stability via ubiquitination and to promote the autophagic response to Mtb infection [29].

The tripartite motif (TRIM) proteins are a large family of ubiquitin ligases with several members playing a role in the regulation of immune responses [30] by interacting with pattern recognition receptors, immune adapter molecules and kinases involved in innate immune signaling pathways [31, 32]. For example, regarding Mtb, TRIM27 functions as a potential restriction factor suppressing the intracellular bacterial survival by enhancing host immune-inflammatory responses and apoptosis mediated by JNK/p38 pathways [33]. Conversely, TRIM25 expression promotes Mtb survival in macrophages possibly by activating the p38 MAPK pathway and suppressing the p65 NF-kB pathway [34].

In addition, several members of TRIM family have been shown to play their roles by modulating the autophagic response by acting at different steps of this process, ranging from upstream signaling pathways and autophagosome formation to cargo recognition and transcriptional regulation of autophagy genes [35]. TRIM5α, 6, 16, 20, 21, 32, 49, and 50 associate with ULK1 and/or BECLIN 1 to promote their activation [31, 36–39]. Moreover, TRIM28, TRIM37, and TRIM19 control autophagy by targeting upstream signaling pathways, such as those regulated by AMPK and mTOR [40–42]. Instead, TRIM22 promotes autophagy by regulating LC3 and BECLIN 1 gene expression through NF‐κB [43]. There is also a subset of TRIM proteins, like TRIM17 or TRIM59, that negatively regulates the autophagy initiation by preferably acting on the BECLIN 1 complex [44, 45].

An important autophagic contribution of TRIM proteins to the innate immune response is their ability to bind pathogens structures and deliver them to the autophagosomes for degradation [35]. Regarding Mtb infection, TRIM-mediated recognition occurs indirectly by binding to galectins that are recruited to bacteria-containing damaged phagosomes, as in the case of TRIM16, which binds to galectin 3 and promotes Mtb ubiquitination and autophagosome engulfment [38].

In this work, we aimed at broadening our knowledge of the role of TRIM proteins in the intracellular response to Mtb infection by combining a transcriptomic (RNA-seq) analysis of TRIM expression in primary macrophages and the effect of ectopic expression of TRIMs on Mtb infection using THP1 macrophage cells. This screening identified TRIM32 as a novel player that is involved in the intracellular immune response to Mtb infection by promoting xenophagy.

## Results

### Modulation of TRIM expression levels in macrophage-derived monocytes infected with Mtb or BCG

**Fig. 1:**
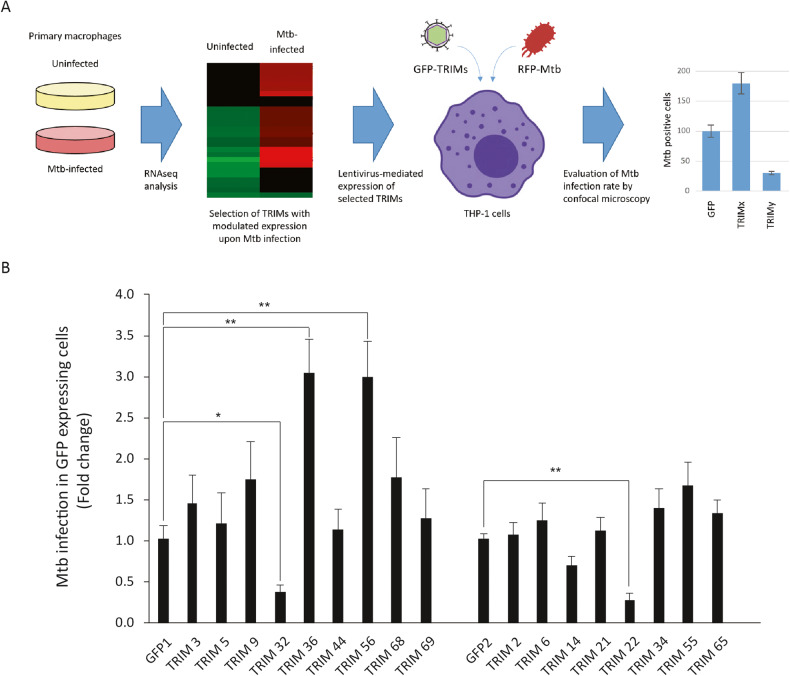
TRIM genes modulated upon Mtb infection. **A** Experimental scheme: human monocyte-derived macrophages purified from peripheral blood mononuclear cells from healthy donors were infected with the Mtb strain H37Rv and transcriptomic changes evaluated after 24 h by RNA-seq. We have identified 17 *TRIM* genes, whose levels are modulated upon infection. cDNAs of identified TRIMs were cloned in a lentiviral vector in frame with the coding sequence for the Green Fluorescence Protein (GFP). Lentiviral particles were produced and used to stably transduce the monocyte cell line THP-1 cells. GFP TRIM expressing cells were differentiated to macrophages using PMA and infected with Mtb H37Rv DsRed. Twenty-four hours post infection, cells were fixed and the percentage of Mtb-infected cells among those positive for GFP were evaluated by confocal microscopy. **B** GFP TRIM expressing THP-1 cells were infected with Mtb H37Rv DsRed. Twenty-four hours post infection, cells were fixed and the percentage of Mtb-infected cells among those positive for GFP were evaluated by confocal microscopy. Ectopic expression of *TRIM22* and *TRIM32* reduces the percentage of Mtb-infected THP1, while *TRIM36* and *TRIM56* have the opposite effect. *P < 0.05, **P < 0.01.

To evaluate the role of TRIM ubiquitin ligases in regulating the innate immune response to Mtb, we analyzed the modulation of their expression levels by RNA-sequencing analysis. Human monocyte-derived macrophages (hMDM) purified from peripheral blood mononuclear cells (PBMC) from healthy donors were in vitro infected with Mtb strain H37Rv or BCG and transcriptomic changes evaluated after 24 h by RNA-seq (Fig. 1A). Experiments were performed with hMDM isolated from three different donors. Differential expression analysis showed that, among 55,766 transcripts identified, 1043 genes were significantly deregulated (fold change≥1.3; *p* ≤ 0.05) by BCG and 2219 genes by Mtb (Table S1). Among them, we identified 17 distinct *TRIM* genes, whose levels were modulated upon infection (Table S2). In particular, Mtb infection was associated to a 2.8-fold increased expression of *TRIM9* and >1.3 fold reduction of *TRIM2, 3, 5, 6, 14, 22, 32, 34, 36, 44, 68, 69* levels when compared to uninfected cells, while BCG increased the expression of *TRIM 5, 22, 55, 56* and decreases that of TRIM2. Comparison of TRIM levels in Mtb- versus BCG-infected cells showed an increased *TRIM9* expression in Mtb vs BCG infected cells, while the levels of *TRIM2, 5, 14, 21, 22, 34, 44, 55, 65, 69* were decreased.

### Identification of TRIM genes that control Mtb replication in THP-1 cells

To elucidate if the *TRIM* genes that are modulated during Mtb/BCG infection play a role in the macrophage antibacterial response, we analyzed the effect of lentivirus-mediated *TRIM* ectopic expression on Mtb infection of THP-1 monocyte cell line. This approach was chosen because most of the modulated *TRIMs* were downregulated during Mtb infection. cDNAs of selected TRIMs were cloned in a lentiviral vector in frame with the coding sequence for the Green Fluorescence Protein (GFP). Lentiviral particles were produced and used to stably transduce THP-1 cells. GFP TRIM-expressing cells were in vitro differentiated to macrophages using PMA and infected with Mtb H37Rv DsRed. Twenty-four hours post in vitro infection, cells were fixed and the percentage of Mtb-infected cells among those positive for GFP were evaluated by confocal microscopy. We found that the ectopic expression of *TRIM22* and *TRIM32* reduces the percentage of Mtb-infected THP1 compared to GFP control cells, while *TRIM36* and *TRIM56* have the opposite effect (Fig. 1B). Interestingly, one of the two *TRIMs* that we found to restrict Mtb replication, *TRIM22*, was previously reported to participate to the host response to viral and bacterial infection [46], including Mtb [43], thus corroborating the reliability of the screening results. We therefore focused on *TRIM32*, since evidences on its contribution on Mtb infection have not been reported so far.

### TRIM32 potentiates the autophagy response in Mtb-infected THP-1 macrophages

**Fig. 2:**
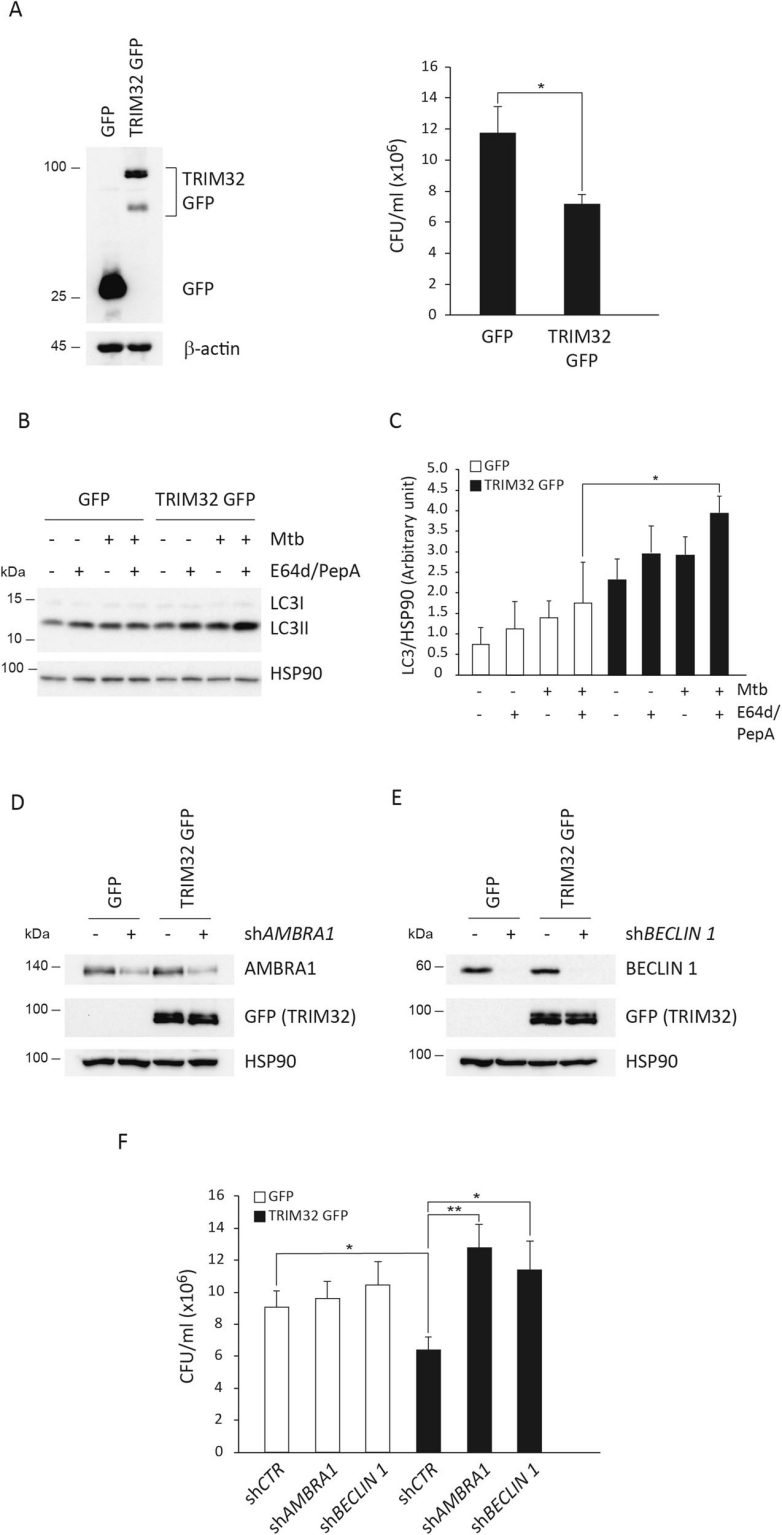
TRIM32 regulates bacterial survival and autophagy in Mtb-infected macrophages. **A** TRIM32-GFP and control GFP expressing THP-1 were differentiated for 24 h with PMA and infected with the Mtb strain H37Rv. Ectopic expression of TRIM32 was monitored by western blot using an anti-GFP antibody (Left Panel). Four days after infection cells were lysed to measure the number of viable bacteria by plating for determining CFU (Right Panel). Values are expressed as a mean of three independent experiments; *P < 0.05. **B** TRIM32-GFP and control GFP expressing THP-1 were differentiated for 24 h with PMA and infected with Mtb strain H37Rv for 24 h or left uninfected. Two hours before lysis, cells were incubated with the lysosome inhibitors E64d and Pepstatin A (E64d/PepA), as indicated. LC3 levels were analyzed by western blot. HSP90 was included as a loading control. The graph reporting means ± SD of LC3-II/HSP90 values from three independent experiments is shown in **C** *P = < 0.05. A.U.: Arbitrary Units. **D**, **E** TRIM32-GFP and control GFP expressing THP-1 were transduced with control shRNA, shAMBRA1 (**D**) or shBECLIN1 (**E**). AMBRA1, BECLIN 1, and TRIM32-GFP levels were analyzed by western blot; HSP90 was included as a loading control. **F** TRIM32-GFP and GFP control cells silenced for *BECLIN 1* or *AMBRA1* were infected with Mtb H37Rv and, 4 days after infection, cells were lysed to measure the number of viable bacteria by CFU counting (shCTR: cells infected with unrelated shRNA lentivirus). Values are expressed as a mean of three independent experiments. *P = < 0.05, **P = < 0.01. Full western blots are reported in Supplemental Fig. 2A.

First, we validate the ability of TRIM32 to restrict Mtb infection by a colony forming units (CFU) counts, the gold-standard for quantifying viable Mtb. As shown in Fig. 2A, a significant reduction in the number of CFU were observed in TRIM32 expressing THP-1 when compared to GFP expressing THP-1 upon infection with Mtb for 4 days.

To elucidate how TRIM32 affects Mtb infection, we started by analyzing whether its ectopic expression results in increased autophagy levels in THP-1 cells, since we and others have previously demonstrated that TRIM32 is involved in the regulation of autophagy [47–49]. GFP- and TRIM32-transduced THP-1 cells were infected with Mtb and autophagy flux was analyzed upon 24 h by comparing the levels of the autophagosome protein LC3-II in the presence or absence of lysosome inhibitors. Immunoblot analysis showed a significant increase in the rate of LC3-II degradation in TRIM32-expressing cells following infection with Mtb (Fig. 2B, C).

### Autophagy inhibition rescues Mtb clearance in TRIM32 expressing macrophages

These results prompted us to assess if autophagy was required for the ability of TRIM32 to restrict Mtb infection. To this aim, GFP and TRIM32 expressing THP-1 were silenced for the expression of two upstream autophagy genes, BECLIN 1 and AMBRA1, infected with Mtb and bacterial replication analyzed by CFU counts 4 days post infection. As shows in Fig. 2D–F, restriction in intracellular Mtb replication in TRIM32 THP-1 was abolished when the expression of BECLIN 1 and AMBRA1 was inhibited, indicating that TRIM32 potentiates the autophagic response in Mtb-infected cells. We also verified that the observed rescue was not due to a reduction of TRIM32 expression in autophagy gene silenced cells (Fig. 2D, E).

### Increased ubiquitination and autophagosomal engulfment of Mtb in TRIM32 expressing THP1

To confirm the hypothesis that the ubiquitin ligase TRIM32 is involved in xenophagy induction in Mtb-infected cells, we analyzed three sequential events required for the autophagosomal engulfment of Mtb: (i) ubiquitination of Mtb (or Mtb-containing structures); (ii) recruitment of ubiquitin-binding autophagy adapters to Mtb; (iii) recruitment of the autophagosomal markers to Mtb.

**Fig. 3:**
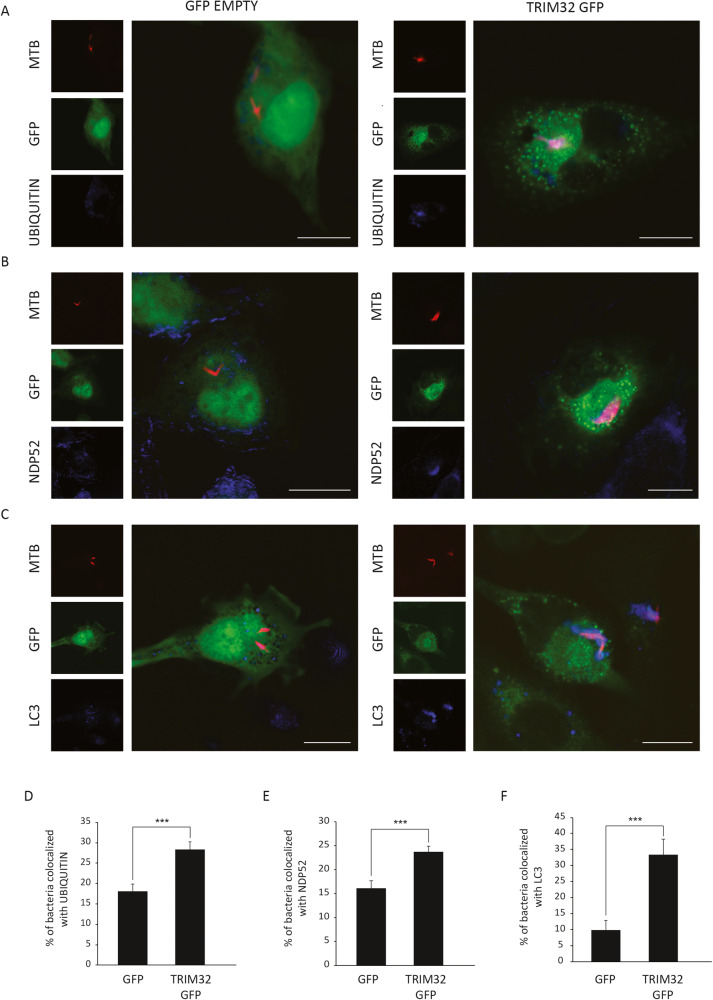
Analysis of Mtb colocalization with LC3, NDP52, and Ubiquitin in TRIM32 expressing cells. TRIM32-GFP (right) and control GFP (left) expressing THP-1 were infected with Mtb H37Rv DsRed. After 24 h cells were fixed and analyzed for Ubiquitin (**A**) or NDP52 (**B**) or LC3 (**C**) localization with Mtb DsRed, by confocal microscopy using specific antibodies. Merging of the three fluorescence signals is shown in the large panels. Scale bar, 10 μm. Colocalization rate was calculated using the ImageJ software. Graphs reporting the quantification of the experiments in **A**–**C**, are shown in **D**–**F**, respectively. The results represent the mean ± SEM of three independent experiments (***P < 0.001).

To this aim, we carried out a colocalization analysis of Mtb DsRed with: (i) ubiquitin (Fig. 3A, D); (ii) the ubiquitin-binding autophagy adapter NDP52 (Fig. 3B, E), which is also recruited to Mtb upon K63-linked polyubiquitination mediated by Parkin [24]; and (iii) the autophagosome marker LC3 (Fig. 3C, F) in GFP and TRIM32 expressing THP-1 24 h post infection by confocal microscopy. Remarkably, all analyzed autophagy markers showed increased colocalization with Mtb in TRIM32 expressing cells compared to controls, confirming that TRIM32 promotes the Mtb clearance mediated by autophagy. Consistent with the involvement of TRIM32 in bacterial ubiquitination, we observed a mean of 10% colocalization of GFP-TRIM32 signal, mainly characterized by dotted structures, with Mtb (Fig. S1), a percentage in line with what reported for Parkin in Mtb-infected macrophages [24].

### TRIM32 is required for triggering an efficient autophagy response to Mtb infection in THP-1 macrophages

**Fig. 4:**
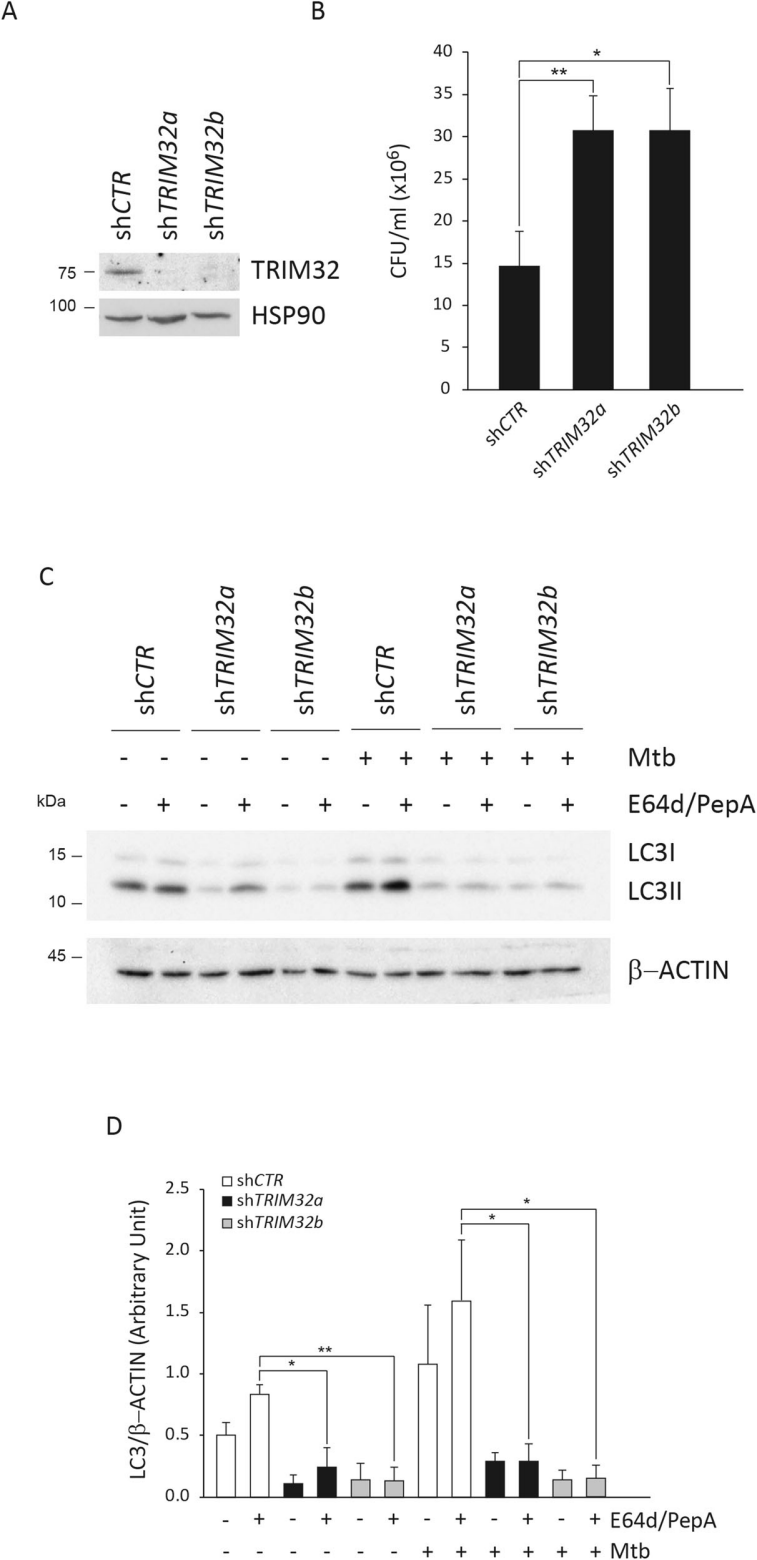
TRIM32 promotes autophagy in Mtb-infected macrophages. **A** THP-1 cells were transduced with control short hairpin RNAs (shRNA) and two independent lentiviral different (sh*Trim32a* and sh*Trim32b*) and silencing verified by western blot using a TRIM32 antibody. **B** Cells described in **A** were infected with the Mtb H37Rv and, 4 days after infection, cells were lysed to measure the number of viable bacteria by CFU counting. Values are expressed as a mean of three independent experiments. *P < 0.05, **P < 0.01. **C** sh*CTR*, *shTRIM32a* and *shTRIM32b* THP-1 cells were infected with Mtb H37Rv for 24 h. Two hours before lysis, cells were incubated with the lysosome inhibitors E64d and pepstatin A (E64d/PepA), as indicated, to evaluate autophagic flux. LC3 levels were analyzed by western blot. β-Actin was included as a loading control. The graph reports means ± SD of LC3-II/β-Actin values from three independent experiments is shown in **D**; *P < 0.05. **P < 0.01. A.U. arbitrary units. Full western blots are reported in Supplemental Fig. 2B.

Based on the results obtained with THP-1 cells ectopically expressing TRIM32, we investigated the contribution of endogenous TRIM32 in the control of Mtb replication. *TRIM32* expression was downregulated by using two different lentiviral short hairpin RNAs (*shTRIM32a* and *shTRIM32b*) in THP1 (Fig. 4A), infected with Mtb and bacterial growth analyzed by CFU counts 4 days after infection. Notably, Mtb replication is significantly increased in *TRIM32* silenced THP-1 (Fig. 4B), indicating that TRIM32 contributes to the control of Mtb replication in macrophages.

**Fig. 5:**
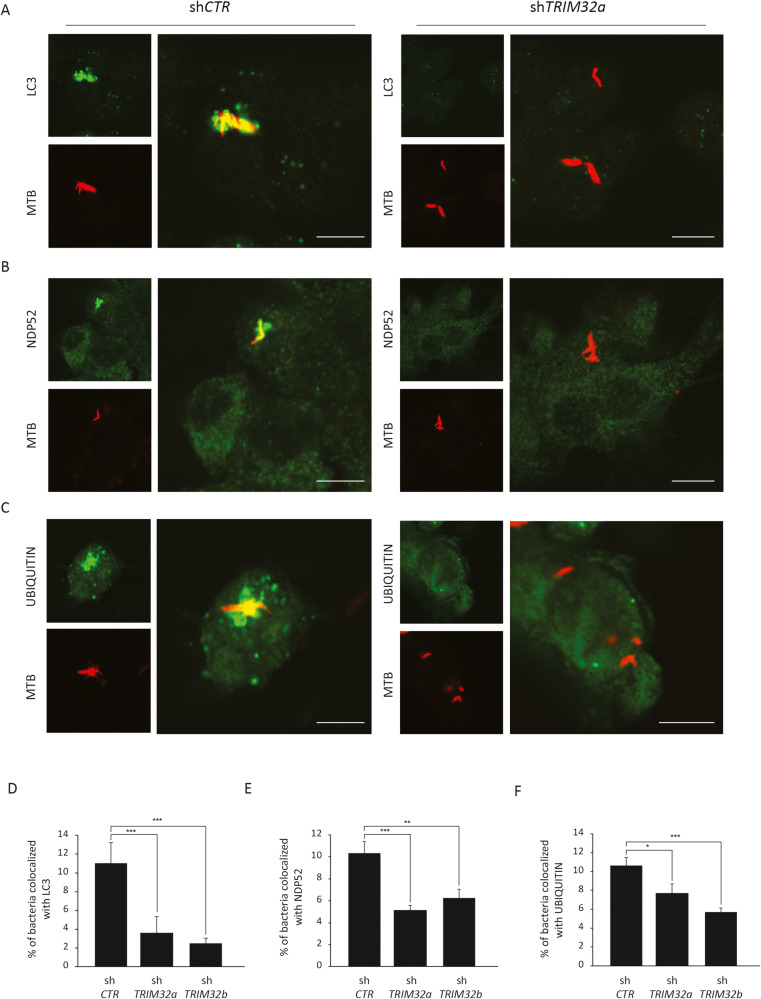
Analysis of Mtb colocalization with LC3, NDP52 and Ubiquitin upon TRIM32 down-regulation. sh*TRIM32* (left) and sh*CTR* (right) THP-1 were infected with Mtb H37Rv DsRed. Cells were fixed and analyzed for LC3 (**A**), NDP52 (**B**), or Ubiquitin (**C**) localization with Mtb H37Rv DsRed, by confocal microscopy, using specific antibodies. Merging of the two fluorescence signals is shown in the large panels. Scale bar, 10 μm. Colocalization rate was calculated using the ImageJ software. Graphs reporting a quantification of the experiments in **A**–**C**, are shown in **D**–**F**, respectively. The results represent the mean ± SEM of three independent experiments (*P ≤ 0.05, **P ≤ 0.01, ***P < 0.001).

Then, we assessed if TRIM32 is required for promoting the autophagic response during Mtb infection by analyzing LC3-II levels by immunoblotting in Mtb-infected cells, in the presence or absence of lysosome inhibitors. As shown in Fig. 4C, D, autophagy levels are critically reduced when *TRIM32* expression is inhibited by two lentiviral shRNAs both in basal conditions and upon Mtb infection, indicating that TRIM32 is required to sustain the autophagic activity in THP-1 cells. Consistently, colocalization of Mtb with LC3-positive autophagosomes is significantly reduced (Fig. 5A, D). Furthermore, we analyzed if TRIM32 deficiency affects early steps of xenophagy i.e.: Mtb ubiquitination and the recruitment of autophagy adapters by confocal microscopy. Confocal analysis showed that colocalization of Mtb with ubiquitin (Fig. 5B, E) and NDP52 (Fig. 5C, F) is drastically reduced in cells silenced for *TRIM32*.

**Fig. 6:**
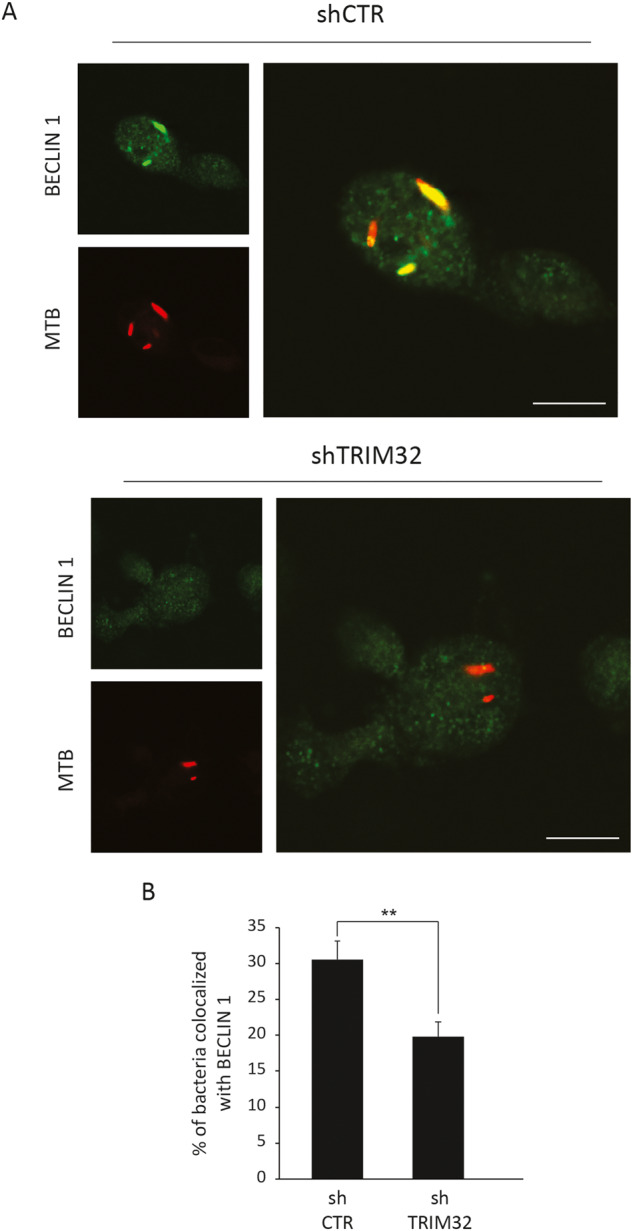
TRIM32 silencing results in an impaired recruitment of BECLIN 1 to intracellular Mtb. **A** sh*TRIM32* (lower panel) and shCTR (upper panel) THP-1 were infected with Mtb H37Rv DsRed. Cells were fixed and analyzed for BECLIN 1 localization with Mtb H37Rv DsRed by confocal microscopy using specific antibodies. Merging of the two fluorescence signals is shown on the large panels. Scale bar, 10 μm. Colocalization rate was calculated by ImageJ software. A graph reporting a quantification of the experiments is shown in **B**. The results represent the mean ± SEM of three independent experiments (**P < 0.01).

Finally, based on the ability of TRIM32 to stimulate the activity of ULK1 and BECLIN 1, we assessed if the impaired autophagy response observed in *TRIM32*-silenced macrophages is associated to a reduced recruitment of the autophagy core machinery to intracellular Mtb [47, 50]. By confocal analysis, we observed, in control cells (shCTR), an extensive translocation of BECLIN 1 on Mtb positive structures (Fig. 6), while ULK1 colocalization was very limited (data not shown), suggesting that ULK1 does not translocate to Mtb or this interaction is highly dynamic. Notably, in *TRIM32*-silenced cells (*shTRIM32*) the colocalization of Mtb with BECLIN 1 (Fig. 6) was significantly reduced.

Altogether, these results indicate that TRIM32 mediates the recruitment to intracellular Mtb of the autophagy machinery required for autophagosome formation and cargo engulfment.

## Discussion

This study shows that the ubiquitin ligase TRIM32 plays an important role in the regulation of xenophagic response to Mtb infection. We have initially identified TRIM32 in an RNA-seq analysis aimed at identifying TRIM genes whose expression is modulated during ex vivo infection of human primary macrophages with Mtb or BCG. This screening identified 17 TRIMs, of which TRIM32, together with TRIM22, showed the largest extent of downregulation in Mtb-infected cells. We have then characterized the role of the identified TRIMs in the host response to Mtb infection by ectopic expression approaches in THP-1 cells and identified TRIM32, together with TRIM22, as TRIM proteins with anti-Mtb replication activity. Further characterization of the molecular mechanisms by which TRIM32 controls Mtb replication showed that TRIM32 induces xenophagy in Mtb-infected cells and autophagy core machinery proteins are required for the antibacterial activity of TRIM32. In addition, using both ectopic expression and RNA interference approaches, we showed that TRIM32 contributes to xenophagy by promoting both the recruitment of BECLIN 1 to Mtb for autophagosome formation and the autophagosome engulfment of Mtb-mediated by ubiquitination.

TRIM32 is a gene mutated in limb-girdle muscular dystrophy 2H, a late-onset autosomal recessive myopathy [51]. TRIM32 is ubiquitously expressed and several evidences point at the role of TRIM32 in regulating cellular processes beyond muscle cells. TRIM32 was originally identified as an interactor of HIV-1 TAT protein. A role of TRIM32 in the innate immunity has been reported in response to viral infection, as in the case of influenza and Herpes Simplex Viruses [52, 53]. In this regard, TRIM32 has been identified as an important regulator of the cGAS–STING pathway of cytosolic DNA sensing [54].

Multiple roles of TRIM32 in the regulation of autophagy have recently emerged. We have shown that the ubiquitin ligase activity of TRIM32 regulates upstream autophagy events in muscle cells upon atrophy induction to protect them from excessive ROS production and consequent induction of the atrophic genes [47]. TRIM32 stimulates autophagy by interacting with ULK1 and AMBRA1 and stimulating ULK1 kinase activity through the formation of unanchored K63-linked polyubiquitin chains. TRIM32 has also been reported to act further downstream in the autophagy process. It positively regulates p62/SQSTM1 activity upon nutrient deprivation, favoring p62 dot formation and autophagosome sequestration via monoubiquitination [49]. Moreover, TRIM32 interacts with TRIF, a critical adapter protein of Toll-like receptors 3/4, and mediates its autophagic degradation in an E3 activity-independent manner by binding to the autophagy cargo receptor TAX1BP1 [48].

Our results suggest that TRIM32 may also act at different steps in xenophagy. TRIM32 silenced cells show a significant decrease in the ubiquitination of Mtb, a prerequisite for the binding of autophagy cargo receptors and autophagosome engulfment. Previous studies have identified the ubiquitin ligases SMURF1 and PARKIN responsible for Mtb ubiquitination and autophagy engulfment [23, 24], highlighting how Mtb ubiquitination could be mediated by the cooperative activity of various ubiquitin ligases. It remains to be elucidated whether TRIM32 is required for the xenophagic activity of SMURF1 and PARKIN or acts independently of them. In addition to a direct role in Mtb ubiquitination, TRIM32 may also facilitate the autophagosome engulfment of Mtb by acting directly as a cargo receptor, as in the case of TRIF [48], or by stimulating the activity of SQSTM1 family proteins through monoubiquitination [49]. Moreover, we found that the inhibition of TRIM32 expression impairs the translocation of BECLIN 1 to Mtb-positive structures, suggesting that TRIM32 is involved in the activation of the core machinery for autophagosome formation in pathogen proximity. In this regard, based on the previously reported role of TRIM32 in cGAS/STING-activated signaling pathways [54], TRIM32 could be involved in transducing pathogen recognition signaling to the autophagy core machinery upon Mtb infection.

In conclusion, our experimental approaches led to the identification of TRIM32 as a novel protein playing a role in the intracellular response to Mtb infection by potentiating autophagy-mediated clearance, whose expression is downregulated in Mtb-infected cells. These results candidate TRIM32 as a novel immune response regulator, whose impairment of expression or activity may be evaluated as a biomarker for TB stages stratification or as a target for host directed therapies. Noteworthy, TRIM32 expression was recently reported to be decreased in leukocytes from TB disease patients in comparison with healthy controls and subjects with TB infection [55].

## Materials and methods

### Cell culture

Macrophage-derived monocytes (MDM) were obtained as described previously [19]. THP-1 cell line (ATCC TIB-202) were cultured in RPMI 1640 (Sigma-Aldrich, R8758) supplemented with 10% of inactivated fetal bovine serum (Gibco, 10270), 2 mM l-glutamine, and 1% penicillin–streptomycin solution (Sigma-Aldrich, G7513/P0781) and maintained in a humidified atmosphere containing 5% CO2 at 37 °C. Cells were screened for mycoplasma contamination by PCR (ABMgood, G238). THP-1 cells were induced to differentiation in RPMI 1640 containing 20 ng/ml of phorbol 12-myristate 13-acetate (PMA, Sigma-Aldrich) for 2 days to obtain THP-1 macrophages. Depending on the experimental requirements, different amounts of cells were used. In details, for the western blot experiments, Thp-1 cells were seeded in 6-well flat bottom tissue culture plates (1 × 10^6^ cells/well, 2 mL/well; Corning). For the confocal experiments, THP-1 cells were seeded in glass slide chambers (Nunc, Lab-Tek) (5 × 10^5^ cells/well, 1 mL/well). For the colony forming unit (CFU) experiment, THP-1 cells were seeded in 24-well flat bottom tissue culture plates (5 × 10^5^ cells/well, 1 mL/well; Corning). For RNA-seq experiments macrophage-derived monocytes were seeded in 6-well flat bottom tissue culture plates (3 × 10^6^ cells/well, 2 mL/well; Corning). Autophagy was evaluated 24 h after infection. To assess the autophagy flux, the lysosomal inhibitors E64d/Pepstatin A (5 µg/mL Santa Cruz Biotechnology, sc-201280A and sc-45036), were added 2 h before lysis.

### Bacteria

The *M. tuberculosis* strain H37Rv, *M. tuberculosis* strain H37RvDsRED, and M. *bovis* Bacillus Calmette et Guerin (BCG) were cultured at the Fondazione Policlinico Gemelli IRCCS, Università Cattolica del Sacro Cuore, as previously described [56]. In brief, the strains were grown in Middlebrook 7H9 (BD NY) supplemented with 10% (vol/vol) oleic acid-albumin-dextrose-catalase (OADC; BD 212240), with 0.2% glycerol (Sigma-Aldrich G7757) and 0.05% Tween 80 (Sigma-Aldrich P8074) at 37 °C. Mycobacterial cultures were harvested at late log phase, glycerol was added at 20% final concentration, and 1-ml aliquots stored at −80 °C. All experiments with *Mtb* strains were carried out in biosafety laboratory level 3 (BSL-3), following standard safety procedures. Macrophages were infected with H37Rv or H37Rv Ds-red or BCG at a multiplicity of infection (MOI) of 5:1 for RNA-Seq analysis, for western blot analysis and CFU and at a MOI of 2:1 for confocal experiments. Two hours after the in vitro infection, macrophages were washed once with phosphate-buffered saline (PBS) and then fresh medium was added.

### Colony-forming unit assay

To determine the intracellular bacterial load, CFUs of infected macrophages were measured in triplicate and determined at 0 h and 4 days post-infection, as described [57]. Briefly, infected cell cultures were lysed in PBS 0.1% Triton X-100 (Sigma-Aldrich, T9284) and the serial dilution was prepared in PBS 0.05% Tween 80 (Sigma-Aldrich P8074) Fifty-microliter aliquots of each dilution were plated on 7H11/ OADC (BD) agar plates. Plates were incubated for 3 weeks.

### Total RNA isolation and RNA-seq analysis

RNA was extracted from BCG and H37Rv infected MDM cells, at 24 h p.i. and from non-infected cells as control, using TRIzol reagent (Invitrogen, 15596-018) according to manufacturer’s guidelines. RNA integrity was evaluated by using the Agilent 2100 Bioanalyzer (Agilent Technologies). Next-generation sequencing experiments were performed by Genomix4Life S.r.l. Indexed libraries were prepared from purified RNA with the TruSeq Total Stranded RNA Sample Prep Kit (Illumina) according to the manufacturer’s instructions. Libraries were quantified using the Agilent 2100 Bioanalyzer (Agilent Technologies). Index-tagged samples were equimolar and the overall concentration was 2 nM. The pooled samples were subject to cluster generation and sequenced using an Illumina HiSeq 2500 System (Illumina) in a 2 × 100 paired-end format at a final concentration of 8 pmol. The resulting short reads were aligned against the GRCh38.p14 genome assembly, using STAR (ver. 2.6.1a). Piled-up reads were counted with htseq-count. Read-count normalization and comparisons were performed using the edgeR R package. Genes were considered differentially expressed between groups if their expression values significantly differed by ≥ 1.3 folds. Correction for multiple tests was achieved by the Benjamini–Hochberg procedure. The significance threshold was set to 0.05.

### Plasmids and cloning

TRIM ORFs were PCR amplified from Genscript vector (TRIM2 OHu30750D, TRIM6 OHu03491D, TRIM34 OHu13090D, and TRIM65 OHu11804), from Sino Biological vector (TRIM14 HG24800-UT, TRIM22 HG19229-UT), from Proteo Genix vector (TRIM21 ID 6737, TRIM36 ID 55521). TRIM5 and TRIM32 were amplified from pcDNA3.1TRIM5 vector and pcDNA3.1TRIM32 vector, respectively, and TRIM 3, 9, 44, 55, 56, 68, and 69 were PCR amplified from a pool of cDNA from Hela, HEK293T, and SHSY5Y cells. All the TRIMs were cloned into a lentiviral pBOB-GFP (Addgene #12337) vector using appropriate oligonucleotides followed by in-frame insertion into the vector.

### Lentiviral transduction

Lentiviral production was performed using 293T as previously described [58] and THP1 cells infected using 50 μL of viral suspension in a medium supplemented with 4 μg/ml polybrene (Sigma-Aldrich, TR-1003) for 8 h. For stable human TRIM32 RNA interference, two lentiviral TRIM32 shRNA–targeting pLKO.1 plasmids were used (TRCN0000273103 and TRCN0000273176; Sigma-Aldrich). For stable human AMBRA1 mRNA interference, a lentiviral AMBRA1 mRNA–targeting pLKO.1 plasmid was used (TRCN0000168652; Sigma-Aldrich). For stable human BECN-1 mRNA interference, a lentiviral BECN-1 mRNA–targeting pLKO.1 plasmid was used (TRCN0000299864; Sigma-Aldrich). A pLKO.1 containing a non-mammalian shRNA was used as a negative control (Sigma-Aldrich).

### Western blot analysis

Cells were collected 24 h post infection and lysed in CelLytic buffer (Sigma-Aldrich, C3228) plus the following protease and phosphatase inhibitors: Protease Inhibitor Cocktail plus (Sigma- Aldrich, P8340), 5 mM sodium fluoride (Sigma-Aldrich, S-7920), 0.5 mM sodium orthovanadate (Sigma-Aldrich, S6508), 1 mM sodium molybdate (Sigma-Aldrich, S-6646), 50 mM 2-chloroacetamide (Sigma-Aldrich, C0267), 2 mM 1,10-phenanthroline monohydrate (Sigma-Aldrich, 320056), and 0.5 mM phenylmethylsulfonyl fluoride (Sigma-Aldrich, P7626). Proteins were separated on SDS PAGE gels and electroblotted onto nitrocellulose (Whatman Amersham, 10600041) or PVDF (Millipore, IPVH20200) membranes. Blots were incubated with primary antibodies in 5% nonfat dry milk (Biosigma, 711160) in PBS (Thermo Fisher Scientific, 18912– 0149) plus 0.1% Tween-20 (Sigma-Aldrich, P1379) overnight at 4 °C. Detection was achieved using horseradish peroxidase–conjugated secondary antibodies (anti-goat 705–036-147, anti-rabbit 711–036-152, and anti-mouse 715–036-150, Jackson ImmunoResearch Laboratories) and enhanced chemiluminescence (ECL, Immobilon Classico WBLUC0500 and Immobilon Crescendo Western HRP substrate WBLUR0500, Millipore). Signals were acquired using a ChemiDoc imaging system. The primary antibodies used in this study were rabbit anti-LC3 (Cell Signaling, Danvers, MA), rabbit anti–BECLIN 1 (Santa Cruz Biotechnology, sc-11427 (H-300)), rabbit anti-AMBRA1 (Millipore, ABC131), rabbit anti-TRIM32 (Thermo Fisher Scientific, PA5-22316), mouse anti-HSP90 (Santa Cruz Biotechnology, sc-13119; F-8), mouse anti β-Actin (Santa Cruz Biotechnology, sc-47778 C4) and mouse anti GFP (Santa Cruz Biotechnology, sc-9996 B-2).

### Confocal microscopy

Confocal microscopy analyses were carried out as previously described [59]. In brief, twenty-four hours after infection, cells were fixed with 4% paraformaldehyde (CARLO ERBA Reagents, 387507) in PBS followed by permeabilization with 0.2% triton X-100 (Sigma-Aldrich, T9284) in PBS. Cells were labeled with the primary antibody anti-LC3 (Cosmo Bio Ltd, CTB-LC3-2-IC), anti-NDP52 (Ab-184688, Abcam), anti-Ubiquitin (FK2) (ST1200, Millipore) for 1 h at room temperature and visualized by means of Cy5-conjugated secondary antibodies (Jackson Immunoresearch) or Alexa Fluor-488 secondary antibodies (Thermo Fisher Scientific). Coverslips were mounted in Prolong Gold antifade (P36935, Life Technologies) and examined under a confocal microscope (Leica TCS SP2). Digital images were acquired with the Leica software and studies of colocalization were performed by using appropriated tools of ImageJ software [60]. A minimum of 200 cells per sample experimental condition were counted for triplicate samples per condition in each experiment.

### Statistical analysis

Statistical analysis of confocal microscopy data were carried out using the Mann-Whitney test (independent samples, two-sided, Graph-Pad, San Diego, CA), while CFU and immunoblotting data were analyzed using unpaired, two-tailed Student’s *t* test (Excel software). Values are shown as mean ± standard deviation of at least 3 independent experiments. Densitometric analysis of immunoblots was performed using the Adobe Photoshop software. The control ratio was arbitrarily defined as 1.00. *P*-values < 0.05, <0.01, <0.001 were marked by *, **, ***, respectively. Normal distribution was assumed on the basis of the appearance of the data, since *n* < 5. No statistical method was used to predetermine sample size. The experiments were not randomized. The investigators were not blinded to allocation during experiments and outcome assessment. No exclusion criteria were applied to exclude samples from analysis.

## Supplementary information

Supplemental Table S1

Supplemental Table S2

Supplemental Figure S1

Supplemental Figure S2A, full images of Western blots reported in Figure 2

Supplemental Figure S2B, full images of Western blots reported in Figure 4

Article checklist

## Data Availability

All data generated or analyzed during this study are included in this published article [and its supplementary information files].
