# Supplementary figures and images for: The ubiquitin ligase TRIM32 promotes the autophagic response to Mycobacterium tuberculosis infection in macrophages
Source: Cell Death Dis. 2023 Aug 5;14(8):505. doi: 10.1038/s41419-023-06026-1 (PMC10404268; doi:10.1038/s41419-023-06026-1)

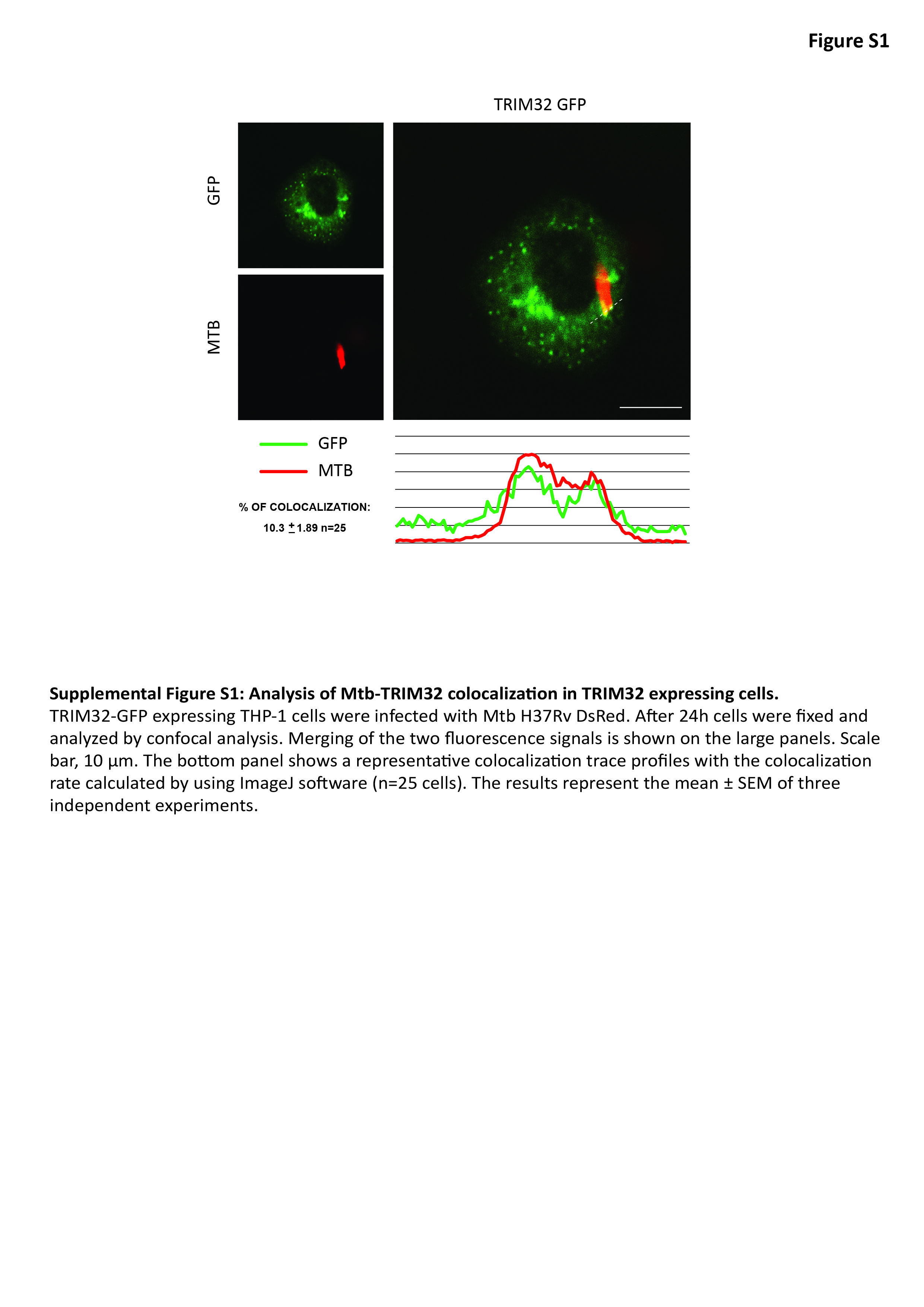

Supplement: Supplementary file 3 — Supplemental Figure S1 [file 41419_2023_6026_MOESM3_ESM.tif]

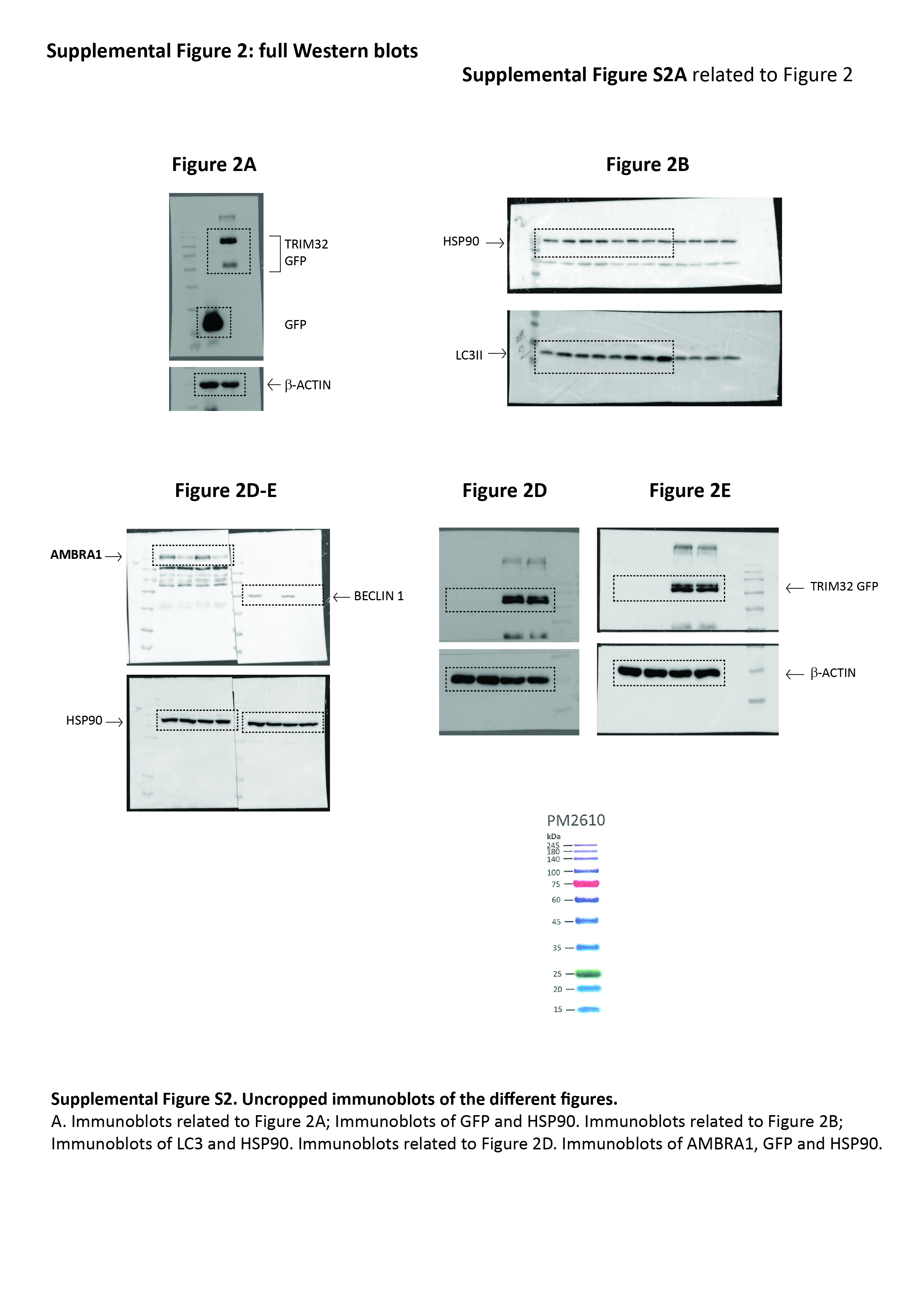

Supplement: Supplementary file 4 — Supplemental Figure S2A, full images of Western blots reported in Figure 2 [file 41419_2023_6026_MOESM4_ESM.tif]

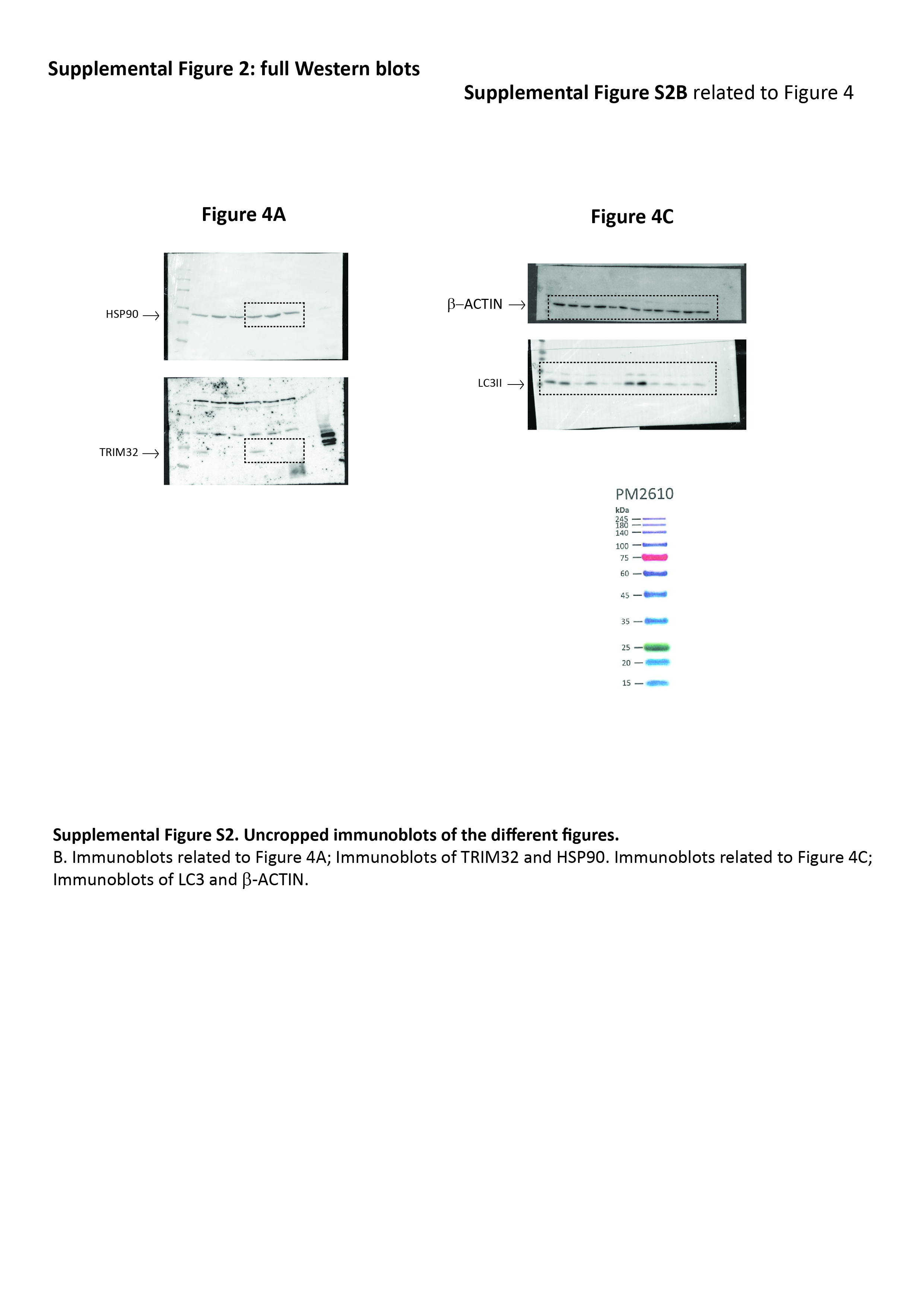

Supplement: Supplementary file 5 — Supplemental Figure S2B, full images of Western blots reported in Figure 4 [file 41419_2023_6026_MOESM5_ESM.tif]
